# Effectiveness and User Experience of Virtual Reality for Social Anxiety Disorder: Systematic Review

**DOI:** 10.2196/48916

**Published:** 2024-02-08

**Authors:** Simon Shahid, Joshua Kelson, Anthony Saliba

**Affiliations:** 1 Faculty of Business, Justice, and Behavioural Sciences Charles Sturt University Bathurst Australia

**Keywords:** social anxiety disorder, social phobia, virtual reality, VR, VR exposure therapy, effectiveness, user experience, safety, usability, acceptability, anxiety, phobia, exposure, systematic, review methods, review methodology, social, psychiatric, mental health, mobile phone

## Abstract

**Background:**

Social anxiety disorder (SAD) is a debilitating psychiatric disorder that affects occupational and social functioning. Virtual reality (VR) therapies can provide effective treatment for people with SAD. However, with rapid innovations in immersive VR technology, more contemporary research is required to examine the effectiveness and concomitant user experience outcomes (ie, safety, usability, acceptability, and attrition) of emerging VR interventions for SAD.

**Objective:**

The aim of this systematic review was to examine the effectiveness and user experience of contemporary VR interventions among people with SAD.

**Methods:**

The Cochrane Library, Emcare, PsycINFO, PubMed, ScienceDirect, Scopus, and Web of Science databases were searched between January 1, 2012, and April 26, 2022. Deduplicated search results were screened based on title and abstract information. Full-text examination was conducted on 71 articles. Studies of all designs and comparator groups were included if they appraised the effectiveness and user experience outcomes of any immersive VR intervention among people with SAD. A standardized coding sheet was used to extract data on key participant, intervention, comparator, outcome, and study design items.

**Results:**

The findings were tabulated and discussed using a narrative synthesis. A total of 18 studies met the inclusion criteria.

**Conclusions:**

The findings showed that VR exposure therapy–based interventions can generally provide effective, safe, usable, and acceptable treatments for adults with SAD. The average attrition rate from VR treatment was low (11.36%) despite some reported user experience difficulties, including potential simulator sickness, exposure-based emotional distress, and problems with managing treatment delivered in a synchronous group setting. This review also revealed several research gaps, including a lack of VR treatment studies on children and adolescents with SAD as well as a paucity of standardized assessments of VR user experience interactions. More studies are required to address these issues.

**Trial Registration:**

PROSPERO CRD42022353891; https://www.crd.york.ac.uk/prospero/display_record.php?RecordID=353891

## Introduction

### Background

Social anxiety disorder (SAD; also known as social phobia) is a psychiatric disorder that is distinguished by a fear of humiliation or negative evaluation by others [1]. Current guidelines highlight the use of cognitive behavioral therapy to treat SAD [2]. This can involve activities such as psychoeducation, relaxation, distraction, cognitive restructuring, exposure therapy, and relapse prevention [2]. Despite the efficacy of cognitive behavioral therapy for SAD treatment [3,4], many who are diagnosed do not go on to seek help [5]. This is partly due to the in vivo (real-life) nature of the exposure therapy used to desensitize and habituate patients to feared situations. It takes significant time, effort, and resources to accurately recreate scenarios that will incite an appropriate level of fear response in social settings [6]. For example, to conduct in vivo exposure therapy with an individual with a fear of public speaking, a therapist would need to gather an audience in an appropriate context (ie, ensuring confidentiality and nonjudgment). Furthermore, social anxiety–provoking environments can be unpredictable, providing therapists with little control and a higher chance that a patient is embarrassed, leading to higher attrition rates [7]. To potentially overcome these issues with delivering in vivo exposure therapy, some researchers have examined the use of virtual reality (VR) technology [7].

### VR Technology

VR technology provides a digital modality to deliver psychological interventions [7,8]. It involves the use of computer hardware and software technology (eg, stereoscopic displays of digital environments) to simulate real-world experiences [7]. For instance, one may enter a virtual environment that mimics a physical environment and could adopt a virtual avatar to interact with this virtual environment [9]. VR was first formulated in the 1960s, with the first commercial device developed in the 1980s [10]. As technology has developed, the quality of images has improved, and costs have been reduced.

VR systems can be divided into 2 categories: immersive and nonimmersive systems [11]. Immersive systems, such as head-mounted displays (HMDs) or cave automatic virtual environments (CAVEs), provide users with a realistic experience of VR environments, whereas nonimmersive systems, such as computer monitors, result in users not feeling as present [12]. Presence in VR refers to the extent of an individual’s perception of being in a particular environment [13]. For VR therapy to be effective, an individual must feel present and immersed in the digital environment [14]. A CAVE system consists of an empty room with multiple screens arranged in a cubelike formation with users wearing stereoscopic glasses and interacting with virtual objects projected onto the screens [15]. Although CAVE systems have the potential to be more immersive than nonimmersive systems, they are expensive and complex to set up, require frequent physical and digital adjustments, and require dedicated personnel [16]. Conversely, a recent systematic review found that current HMDs offer a more immersive experience than CAVEs and are significantly more user-friendly in cost and setup, with a “plug-n-play” setup solution [15].

When integrated with therapy, VR technology can help address factors that influence the success of exposure-based treatments. For instance, VR allows for the creation of controlled digital environments, which enables therapists to predictably customize exposure scenarios to the specific needs and fears of individual clients [7]. VR can also improve accessibility to exposure therapy for individuals who find it logistically challenging or emotionally overwhelming to engage in real-world scenarios [17]. The immersive nature of VR helps bridge the gap between simulated experiences and real-life situations, fostering a sense of presence and engagement that can potentially enhance treatment adherence and effectiveness [17].

### Effectiveness of VR for SAD

Virtual environments and avatars can be used to simulate socially distressing situations for SAD treatment. For example, a study immersed participants with SAD into a computer-generated classroom where they were asked to speak publicly on a topic while a therapist controlled the virtual audience’s reactions according to the stage of therapy [18]. VR environments have also been shown to provide acceptable levels of presence and immersion that are necessary for exposure therapy in youth with social anxiety [19]. Several systematic reviews and meta-analyses have demonstrated the effectiveness of VR exposure therapy (VRET) in the treatment of SAD [6,20-24]. Indeed, researchers have established a large effect size for VRET versus waitlist (*g*=0.90), a medium to large effect size for VRET versus psychological placebo conditions (*g*=0.78) [21], a large overall effect size for VRET (*g*=0.82) [22], and a medium to large effect size for VRET at the 12-month follow-up (*g*=−0.74) [6]. This consistent pattern of symptom reduction can be observed across various contexts, such as participant countries (eg, the United States, France, Israel, and South Korea) and treatment settings (eg, universities, hospitals, and clinics) [6,20-24]. However, although existing reviews have explored VR-based therapy from an effectiveness standpoint (eg, reduction in anxiety symptoms), there are gaps in the literature on evaluating the VR user experience for people with SAD on key concomitant outcomes of safety, usability, acceptability, and attrition in different contexts.

### User Experience of VR for SAD

#### Safety

Studies using VR for workplace training, physical rehabilitation, psychological therapy, and other settings highlight a significant safety issue: simulator sickness [25]. Simulator sickness (otherwise known as VR sickness or cybersickness) [26-28] is characterized by general discomfort, headache, eyestrain, nausea, difficulty concentrating, fatigue, blurred vision, dizziness, and vertigo. On the basis of postural instability theory, simulator sickness is arguably due to VR technology inducing sensory differences in the visual and vestibular systems, which coordinate balance and movement [28-30]. The human body may interpret these disparities as possibly deadly causes (ie, consuming poison) and seek to purge as a result [25]. Consequently, simulator sickness can have a negative impact on participants during VR use and for hours following use [31]. Other aspects of safety include physical injuries from repetitive strain, users colliding with objects in the real world, poor posture, headset discomfort, risk of inducing epileptic seizures, negative mood changes, and infection control [32]. Overall, these issues might put participants at risk of harm or cause them to discontinue using VR. Thus, a comprehensive examination of VR safety for SAD is necessary.

#### Usability

There does not yet appear to be a framework for the evaluation of VR usability in therapy-based applications. Nielsen [33] defines usability as a “quality attribute” that assesses how easy it is to interact with an interface. He highlighted 5 components: learnability (how easy it is for a beginner to use the interface), efficiency (once the user has learned to use the interface, how quickly they can perform tasks), memorability (re-establishing proficiency after a period of absence), errors (frequency, severity, and recoverability of errors), and satisfaction (level of pleasure from using the interface) [33]. Although this framework is applied to website design, it can also be applied to participants’ perceptions of the usability of VR. Furthermore, the application of VR in real-world settings (eg, in a therapy room) would likely be performed by a clinician rather than a specialized technician. It is important to note that usability issues may arise among clinicians. For example, they may give up on the technology if components fail to load or connect to each other. For this reason, this review included both clinician experiences in administering VR-based therapy and client experiences.

#### Acceptability

Acceptability is a crucial consideration when evaluating VR interventions [34]. It involves assessing the degree to which the new intervention and its components are received and aligned with the needs of the target population [34]. For example, a study examining VR use in adults with SAD defined acceptability as a participant’s willingness to use a VR program [35]. They measured acceptability by observing rates of attrition and responses to the following question—“Would you recommend this program to others who might have problems similar to yours?”—and inviting further feedback. Participants’ additional feedback was coded into 2 themes: satisfaction (sense of realism, insight, and utility) and perceived effects of the treatment (impact on anxiety). The findings indicated that VR was considered acceptable by participants on all measures [35]. Nevertheless, although there are recent systematic reviews that have addressed the acceptability of VR use for the general population [36], psychosis [37], panic disorder [38], and posttraumatic stress disorder [39,40], a review of the literature on the acceptability of VR in individuals with SAD does not yet exist based on our current knowledge.

#### Attrition

Attrition, the discontinuation of therapy before treatment completion and resolution of symptoms, can have profound negative effects [41]. These can include the client not fully benefiting from therapy and being discouraged from seeking treatment in the future [41] as well as the effect that this may have on the therapist (eg, loss of revenue, demoralization, and feelings of failure) [42]. A recent meta-analysis of VRET showed significant heterogeneity in attrition rates in the treatment of anxiety disorders, highlighting reasons such as failure to immerse in the virtual environments, simulator sickness, vision complications, and difficulty communicating with a therapist that the participant could not see [43]. A systematic review examining the available literature on rates of attrition of VR-based interventions (both VRET and non-VRET) with participants with SAD does not yet appear to exist based on our current knowledge.

### This Study

This study aimed to systematically identify and review available evidence regarding the effectiveness and user experience (ie, safety, usability, acceptability, and rates of attrition) of VR interventions in the treatment of SAD. The following objectives aided in the provision of a comprehensive and up-to-date account of the empirical status of VR therapy for SAD: (1) provide an overview of the existing literature and identify areas in which further research is needed on the treatment of SAD; (2) assess the potential of using VR as a treatment option for SAD, specifically in terms of effectiveness and user experience; and (3) provide guidance and recommendations for future research regarding the use of VR as a treatment option for SAD.

## Methods

This systematic review was conducted using the PRISMA (Preferred Reporting Items for Systematic Reviews and Meta-Analyses) checklist (Multimedia Appendix 1) [44]. The protocol of this systematic review was prospectively registered in the PROSPERO international database (CRD42022353891).

### Eligibility Criteria

For articles to be included in this systematic review, the study participants needed to be people diagnosed with SAD regardless of age. If a study had a mix of people with and without SAD, that study would be included if subgroup analyses were available on the participants with SAD or if they made up the vast majority (ie, ≥80%). All studies needed to examine direct participant use of a VR intervention, which includes any system that incorporates immersive VR hardware (ie, HMD or CAVE systems). Only studies that were published after 2012 were included as this marked the introduction of widely available commercial HMD hardware such as the Oculus Rift [9]. Such hardware allowed for the delivery of VR experiences comparable with previously expensive commercial setups at a cheaper cost as well as easier accessibility to researchers [45]. Studies with research design comparators of any kind (eg, comparing VR with other non-VR interventions) were eligible for inclusion. All studies were required to report on VR intervention effectiveness and participant user engagement outcomes. This broadly included any standardized or unstandardized measure indicative of usability or acceptability (including attrition rates). Studies of all designs (ie, quantitative, qualitative, and mixed methods) were eligible for inclusion. No studies were excluded based on methodological quality. All the articles needed to be written in English and published in peer-reviewed journals.

### Search Strategy

Prominent scientific research databases were searched between January 1, 2012, and April 26, 2022: Cochrane Library, Emcare, PsycINFO, PubMed, ScienceDirect, Scopus, and Web of Science. The following keywords were used to search the databases: (“virtual reality” or “VR”) and (“social anxiety” or “social phobia”). The reference lists of eligible articles were also searched.

### Article Selection

The search results for all databases were deduplicated, and the remaining article titles and abstracts were scanned. Full-text appraisal was performed on promising articles, and the final study inclusion was agreed upon by the researchers using the eligibility criteria. Divergent views on inclusion were resolved through discussion and mutual agreement.

### Data Extraction

Data from the included studies were extracted by one reviewer (SS) into a standardized coding sheet and then checked by a second reviewer (JK). The data types extracted from eligible papers included the following:

1. Reference source: author surnames, year of publication, and paper title.
2. Sample: country; sample size; and nonidentifiable participant characteristics such as age, sex, and diagnosis.
3. Study design: methodology, comparator trial arms, and measurement points (pretest, midtest, and posttest measurement and follow-up).
4. VR intervention details: intervention program name, purpose of intervention (eg, exposure therapy, cognitive distraction, or relaxation), virtual environment type, hardware (eg, HMD or CAVE system), and treatment length.
5. Effectiveness: standardized measure names, outcomes, and effect sizes.
6. User experience: reported outcomes of intervention safety, usability, acceptability, attrition, and intention-to-treat analyses.

Attrition in this review was defined and measured as the relative number of participants who began using the VR intervention but did not complete measurements during or after the intervention.

### Quality Assessment

This systematic review included randomized controlled trial (RCT) and nonrandomized studies. Therefore, the Mixed Methods Appraisal Tool (MMAT) was used to assess the quality of all the included studies [46]. The MMAT was used as it assesses methodological quality across 5 study categories: RCTs, nonrandomized quantitative studies, quantitative descriptive studies, qualitative studies, and mixed methods studies.

### Data Analysis

A narrative synthesis approach was used in this systematic review. This involved summarizing and explaining the findings using text as a statistical meta-analysis was not possible because of data heterogeneity across the included studies.

## Results

### Study Selection

**Figure 1:**
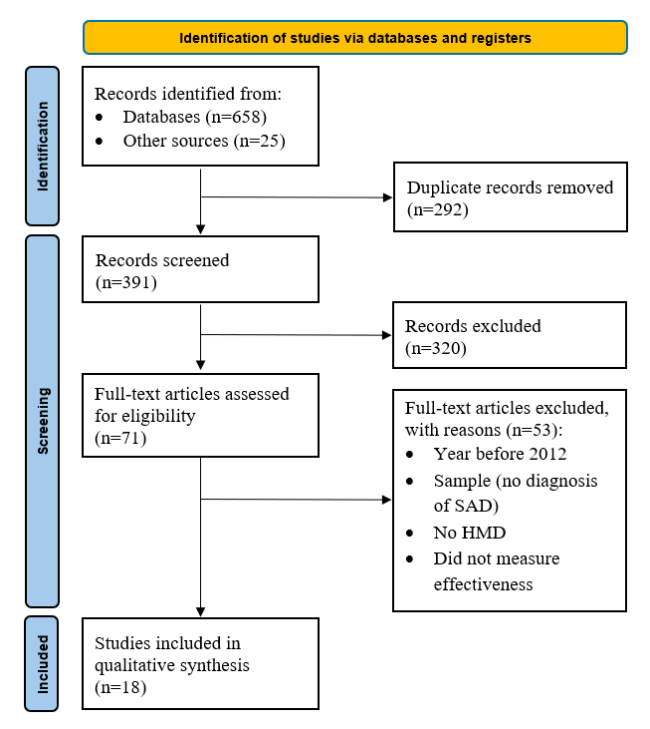
Flowchart of the systematic review search results. HMD: head-mounted display; SAD: social anxiety disorder.

Figure 1 shows that the literature search yielded 683 articles, of which 391 (57.2%) remained after deduplicating citations. Of these 391 records, 18 (4.6%) met the eligibility criteria.

### Participant Characteristics

A total of 808 participants were recruited for the VR studies (Table 1). They were largely from South Korea (n=368), followed by the United States (n=163), the Netherlands (n=60), Canada (n=59), Sweden (n=23), Czech Republic (n=10), Denmark (n=9), and Brazil (n=2). The country of origin was missing for some participants (n=114). It is unclear whether participants were unique in 11% (2/18) of studies conducted by research teams with some of the same researchers [47,48]. The sample sizes ranged from 1 to 115 participants, with a median of 48 participants. Participants’ ages ranged from 18 to 65 years. Participants were mainly female, with an average sample proportion of 51.31% (SD 5.36%; range 0%-77.3%). Most participants were diagnosed with SAD. There were 9 participants with a diagnosis of flight phobia and 8 participants with a diagnosis of acrophobia; however, subgroup analyses were available for the participants with SAD in this study [49]. All participant diagnoses were obtained through clinical interviews delivered in person, by phone, or via videoconferencing. In total, 3 therapists were interviewed in addition to the sample of participants in one study [50].

**Table 1:** Description of participants and research designs in the reviewed studies.

Study	Country	Sample size^a^	Total sample mean age (years; SD)	Total sample age range (years)	Total sample percentage of female participants	Study design	Treatment conditions^b^	Measurements
Anderson et al [51]	United States	97	39.03 (11.26)	19-69	61.9	RCT^c^	EGT^d^: 25; VRET^e^: 25; WL^f^: 25	Pre- and posttest measurement and 3- and 12-month FU^g^
Arnfred et al [50]	Denmark	9	25.4 (6.54)	19-35	66.7	Interview	VRET: 9	Posttest measurement
Bouchard et al [52]	Canada	59	34.5 (11.9)	18-65	72.9	RCT	VRET: 17; in vivo: 22; WL: 20	Pre- and posttest measurement and 6-month FU
Geraets et al [53]	NR^h^	15	34.9 (12.4)	18-65	53.3	Single group	VRET: 15	Pre- and posttest measurement and 6-month FU
Hur et al [54]	South Korea	73	NR	NR	42.6	Case controlled	VRET: 25; HC^i^: 22	Pre- and posttest measurement
Jeong et al [55]	South Korea	115	NR	NR	34.8	Cohort	ET^j^: 52; NT^k^: 43; SE^l^: 20	Pre- and posttest measurement
Kampmann et al [56]	The Netherlands	60	36.9	18-65	63.3	RCT	VRET: 20; WL: 20; iVET^m^: 20	Pre- and posttest measurement and 3-month FU
Kim et al [47]	South Korea	54	23	NR	57.7	Controlled clinical trial	VRET: 22; HC: 30	Pre- and posttest measurement
Kim et al [57]	South Korea	74	NR	19-31	56.9	Longitudinal	VRET: 32; HC: 33	Pre- and posttest measurement
Kim et al [48]	South Korea	52	NR	19-30	NR	RCT	VRS^n^: 24; WL: 28	Pre- and posttest measurement
Kovar [58]	Czech Republic	10	34.6 (11.7)	19-51	50	Nonrandomized parallel comparison trial	Psychotherapy: 5; psychotherapy+VRET: 5	Pre- and posttest measurement
Lindner et al [59]	Sweden	23	40.61 (10.15)	≥18	57	Cohort	VRET: 23	Pre- and posttest measurement
Moldovan and David [49]	NR	32	NR	≥18	46.9	RCT	VRCBT^o^: 16; WL: 16	Pre- and posttest measurement and FU
Perandré and Haydu [60]	Brazil	2	23.5 (4.9)	20-27	0	Case study	VRET: 2	Pre- and posttest measurement and 1- and 3-month FU
Price and Anderson [61]	NR	67	40.31 (11.55)	NR	69	RCT	VRET: 33; EGT: 34	Pre-, mid-, and posttest measurement
Rubin et al [62]	United States	21	NR	18-65	61.9	RCT	VRET: 10; VRET+AGT^p^: 11	Pre- and posttest measurement and 1-week FU
Trahan et al [63]	United States	1	36	NR	0	Case study	VRET: 1	Pre- and posttest measurement
Zainal et al [64]	United States	44	23.3 (9.32)	18-53	77.3	RCT	VRET: 26; WL: 18	Pre- and posttest measurement and 3- and 6-month FU

^a^Refers to the total number of participants in the study.

^b^Refers to the number of participants in each treatment condition.

^c^RCT: randomized controlled trial.

^d^EGT: exposure group therapy.

^e^VRET: virtual reality exposure therapy.

^f^WL: waitlist control.

^g^FU: follow-up.

^h^NR: not reported.

^i^HC: healthy controls.

^j^ET: early termination.

^k^NT: normal termination.

^l^SE: session extension.

^m^iVET: in vivo exposure therapy.

^n^VRS: virtual reality self-training.

^o^VRCBT: virtual reality cognitive behavioral therapy.

^p^AGT: attention guidance training.

### Details of the VR Interventions

All the studies included VR-based exposure therapy. Nearly all the studies (15/18, 83%) tested a unique VR intervention except for the studies by Jeong et al [55] and Kim et al [47,48] (Table 2). VR hardware included standard computers, smartphones, and HMDs. In total, 17% (3/18) of the studies [49,51,61] did not identify the headset brands. The studies used custom-built software that immersed participants in VR environments that simulated social situations increasing in difficulty with audio, video, text, and interactivity. The treatment lengths ranged from 1 to 14 sessions of exposure therapy, with a mode of 8. A total of 17% (3/18) of the studies [53,55,64] terminated the sessions early if habituation occurred before the completion of the sessions. Participant VR use time ranged from 5 minutes to 3 hours per session, and 17% (3/18) of the studies delivered the VR in a single session [49,59,62]. All VR interventions were tested with therapist or facilitator guidance even though 22% (4/18) [47,48,55,64] were designed to be delivered as self-help.

**Table 2:** Details of the virtual reality (VR) interventions.

Study	Virtual environments	Headset	Treatment length (duration)
Anderson et al [51]	Conference room, classroom, and auditorium	—^a^	4 exposure sessions (30 min each)
Arnfred et al [50]	Supermarket, meeting, cafeteria, party, and auditorium	Oculus Go	8 exposure sessions (45 min each)
Bouchard et al [52]	Meeting room, job interview, apartment, coffee shop, neighbors, store, and neutral	eMagin Z800	8 exposure sessions (20-30 min each)
Geraets et al [53]	Street, bus, café, and supermarket	Sony HMZ-T1	14 exposure sessions (40 min each)
Hur et al [54]	College student group	HTC Vive	6 exposure sessions (5-8 min each)
Jeong et al [55]	School, business, and daily life	Samsung Gear VR powered by Oculus	ET^b^ (1-8 exposure sessions); NT^c^ (9-10 exposure sessions); SE^d^ (11-17 exposure sessions)
Kampmann et al [56]	Audience, stranger, clothes shopping, job interview, journalist interview, restaurant, and blind date	nVisor SX	7 exposure sessions (60 min each)
Kim et al [47]	School, business, and daily life	Samsung Gear VR powered by Oculus	8 exposure sessions
Kim et al [57]	College student group	HTC Vive	6 exposure sessions
Kim et al [48]	School, business, and daily life	Samsung Gear VR powered by Oculus	8 exposure sessions
Kovar [58]	Public speaking, telephone call, receiving criticism, job interview, refusal of job offer or unwanted product, and working lunch	HTC Vive	8 exposure sessions
Lindner et al [59]	Board room, conference room, and classroom	Oculus Go	1 exposure session (180 min)
Moldovan and David [49]	Presentation and interview	—	1 exposure session (90 min)
Perandré and Haydu [60]	Food court in shopping center	Oculus Rift	8 exposure sessions
Price and Anderson [61]	Conference room, classroom, and auditorium	—	8 exposure sessions
Rubin et al [62]	Conference room and auditorium	Oculus Rift DK2	1 exposure session (45 min)
Trahan et al [63]	Grocery store	Plastic HMD^e^ bracket for mobile phone	12 exposure sessions (12-15 min each)
Zainal et al [64]	Dinner party and job interview	Pico Goblin VR	8 exposure sessions (25-30 min each)

^a^Brand not reported.

^b^ET: early termination.

^c^NT: normal termination.

^d^SE: session extension.

^e^HMD: head-mounted display.

### Research Designs and Comparators

Table 1 summarizes the research designs and comparators. Nearly half (8/18, 44%) of the studies appraised participant VR use through RCT designs. Comparators included exposure group therapy, in vivo exposure, early and extended termination, attention guidance training using VR, psychotherapy, and waitlist control. All studies (18/18, 100%) had pre- and posttest assessments of user outcomes, although 39% (7/18) also had follow-up assessments, with the longest being 12 months [51].

### Effectiveness Measures and Outcomes

The details of the effectiveness measures and outcomes of VR treatment for SAD are summarized in Table 3. VR treatment effect sizes across all studies that reported them ranged from medium to large. Almost all studies (15/18, 83%) demonstrated a decrease in symptoms following VR treatment.

**Table 3:** Details on social anxiety measures and virtual reality (VR) effectiveness outcomes.

Study and measures		VR effectiveness outcomes
**Anderson et al [51]**		
	PRCS^a^	Significant improvement in confidence as a speaker from before to after treatment (*d*^b^=1.19; *P*=.01), with benefits maintained at the 3- and 6-month follow-ups.
	FNE-B^c^	Significant decrease in fear of negative evaluation from before to after treatment (*d=*0.29; *P*=.01), with benefits maintained at the 3- and 6-month follow-ups.
	BAT^d^	Significant improvement in speech length (*d*=0.78; *P*=.01) and peak anxiety (*d*=0.70; *P*=.02) from before to after treatment.
**Arnfred et al [50]**		
	NSQ^e^	The virtual environments effectively induced immersion and anxiety in some but not all participants with social anxiety disorder.
**Bouchard et al [52]**		
	LSAS-SR^f^	Significant decrease in social anxiety symptoms from before to after treatment compared with waitlist (*P*<.001) that was maintained at the 6-month follow-up.
	BAT	Significant decrease in behavioral avoidance from before to after treatment (*P*<.001).
	SPS^g^	Significant decrease in social phobia from before to after treatment (*P*<.001) that was maintained at the 6-month follow-up.
	SIAS^h^	Significant decrease in social anxiety symptoms from before to after treatment (*P*<.001) that was maintained at the 6-month follow-up.
	FNE^i^	Significant decrease in fear of negative evaluation from before to after treatment (*P*<.001) that was maintained at the 6-month follow-up.
**Geraets et al [53]**		
	SIAS	Significant decrease in social interaction anxiety from before to after treatment (*d*=0.9; *P*=.008) that was maintained at the 6-month follow-up (*d*=1.3; *P*=.003).
**Hur et al [54]**		
	SPS	Significant decrease in social phobia symptoms from before to after treatment (*P*=.005).
	PERS^j^	Significant decrease in negative postevent rumination from before to after treatment (*P*<.001).
**Jeong et al [55]**		
	FNE-B	Significant decrease in fear of negative evaluation from first to last session for the early, normal, and extended termination groups (*P*<.001).
	LSAS	Significant decrease in social anxiety symptoms from first to last session for the normal (*P*<.001) and extended termination groups (*P*=.002).
	SPS	Significant decrease in social phobia symptoms from first to last session for the normal (*P*=.001) and extended termination groups (*P*<.001).
	SIAS	Significant decrease in social interaction anxiety from first to last session for the normal (*P*<.001) and extended termination groups (*P*=.006).
**Kampmann et al [56]**		
	LSAS-SR	Significant decrease in social anxiety symptoms from before to after treatment compared with waitlist (*d*=0.55; *P*=.01) that was maintained at the 3-month follow-up.
	FNE-B	No significant change in fear of negative evaluation compared with waitlist group from before to after treatment or the 3-month follow-up.
	BAT	Significant increase in speech length from before to after treatment compared with waitlist (*d*=0.56; *P*=.02) that was maintained at the 3-month follow-up; however, there was no significant difference in speech performance.
**Kim et al [47]**		
	HADS^k^	Significant decrease in anxiety symptoms from before to after treatment (*P*<.001).
	LSAS-SR	Significant decrease in social anxiety symptoms from before to after treatment (*P*<.001).
	SIAS	Significant decrease in social interaction anxiety from before to after treatment (*P*<.001).
**Kim et al [57]**		
	SPS	Significant decrease in social phobia symptoms from before to after treatment (*P*<.001).
	SIAS	Significant decrease in social interaction anxiety from before to after treatment (*P*<.001).
	FNE-B	Significant decrease in fear of negative evaluation from before to after treatment (*P*=.004).
	KSAD^l^	Significant decrease in social avoidance and distress from before to after treatment (*P*<.001).
	LSAS^m^	Significant decrease in social anxiety symptoms from before to after treatment (*P*=.04).
**Kim et al [48]**		
	HADS	No significant changes in anxiety symptoms from before to after treatment.
	LSAS-SR	Significant decrease in social anxiety symptoms from before to after treatment (*P*<.01).
**Kovar [58]**		
	FNE-B, SPIN^n^, SIAS, SADS^o^, and BAI^p^	Higher average decrease in symptoms on all these measures in the VR treatment group compared with the non-VR treatment group.
**Lindner et al [59]**		
	PSAS^q^	Significant decrease in self-rated public speaking anxiety following the first 3-hour session (*d*=0.77; *P*=.006).
	LSAS-SR	Significant decrease in social anxiety symptoms from before to after treatment (*P*=.001).
	FNE-B	Significant decrease in fear of negative evaluation from before to after treatment (*P*=.04).
**Moldovan and Price [49]**		
	FNE-B	Significant decrease in fear of negative evaluation from before to after treatment (*P*<.05).
	SSPS^r^	Significant decrease in negative self-statements from before to after treatment (*P*<.05).
	LSAS	Significant decrease in social anxiety symptoms from before to after treatment (*P*<.05).
**Perandré and Haydu [60]**		
	SPIN and BAI	Decrease in anxiety symptoms reported on both measures from the pretest measurement to the 3-month follow-up from treatment for both participants.
**Price and Anderson [61]**		
	SSPS	Significant improvements on positive and negative self-statements from before to after treatment (*P*<.01).
	PRCA-SF^s^	Significant decrease in public speaking anxiety from before to after treatment (*P*<.01).
**Rubin et al [62]**		
	PRPSA^t^	Significant decrease in fear of public speaking from before to after treatment (*d*=−1.11) and at the 1-week follow-up (*d*=−1.68).
	LSAS-SR	Significant decrease in general symptoms of social anxiety from before to after treatment (*d*=−0.60) and at the 1-week follow-up (*d*=−2.07).
**Trahan et al [63]**		
	SUDS^u^	No significant change in subjective distress from before to after treatment for the participant (*P*=.21).
	SADS	Score decrease of 52.6% in social anxiety from before to after treatment for the participant.
**Zainal et al [64]**		
	SAD composite^v^	Significant decrease in social anxiety symptoms from before to after treatment compared with the waitlist group (*g*^w^=−4.77; *P*<.001). No significant within-group change at the 3- (*g*=0.12) and 6-month follow-ups (*g*=−0.13).
	MASI^x^	Significant decrease in job interview anxiety from before to after treatment compared with the waitlist group (*g*=−4.17; *P*<.001). No significant within-group change at the 3- (*g*=−0.10) and 6-month follow-ups (*g*=−0.53).

^a^PRCS: Personal Report of Confidence as a Speaker.

^b^Cohen *d* effect size.

^c^FNE-B: brief Fear of Negative Evaluation Scale.

^d^BAT: behavioral avoidance task.

^e^NSQ: nonstandardized questions.

^f^LSAS-SR: Liebowitz Social Anxiety Scale–Self-Report.

^g^SPS: Social Phobia Scale.

^h^SIAS: Social Interaction Anxiety Scale.

^i^FNE: Fear of Negative Evaluation Scale.

^j^PERS: Post-Event Rumination Scale.

^k^HADS: Hospital Anxiety and Depression Scale.

^l^KSAD: Korean Social Avoidance and Distress Scale.

^m^LSAS: Liebowitz Social Anxiety Scale.

^n^SPIN: Social Phobia Inventory.

^o^SADS: Social Avoidance and Distress Scale.

^p^BAI: Beck Anxiety Inventory.

^q^PSAS: Public Speaking Anxiety Scale.

^r^SSPS: Self-Statements During Public Speaking scale.

^s^PRCA-SF: Personal Report of Communication Apprehension–Short Form.

^t^PRPSA: Personal Report of Public Speaking Anxiety.

^u^SUDS: Subjective Units of Distress Scale.

^v^SAD composite: average standardized scores of the Social Phobia Diagnostic Questionnaire and the SIAS.

^w^Hedges *g* effect size.

^x^MASI: Measure of Anxiety in Selection Interviews.

### User Experience With the VR Interventions

The average attrition rate was 11.36% across all studies in the active VR treatment phase, with a range of 0% to 45.2% (Table 4). A total of 22% (4/18) of the studies reported the use of an intention-to-treat analysis. To measure VR user experience, 56% (10/18) of the studies used standardized measures, and 11% (2/18) of the studies used nonstandardized questions. A total of 67% (8/12) of these studies reported positive VR user experience findings in various areas of presence, usability, acceptability, or satisfaction. Low levels of simulator sickness were reported in 75% (3/4) of the studies that used standardized questions; however, 25% (1/4) of these studies reported higher levels of simulator sickness in participants with SAD than in controls without SAD [47]. No other safety issues, such as physical injury, user collision, postural complaints, headset discomfort, seizures, or infection, were reported.

**Table 4:** Virtual reality (VR) interventions and user experience outcomes.

Study	Measures	VR user experience findings	Attrition (%)	ITT^a^
Anderson et al [51]	CSQ^b^	High satisfaction with VR was reported after treatment and maintained at the 12-month follow-up.	5/30 (17)	Yes
Arnfred et al [50]	NSQ^c^	A high level of presence in virtual environments for some participants but not all. There were technical issues with setting up and storing away equipment for the group. Wearing the HMD^d^ in front of strangers was more anxiety provoking than the virtual environments for some participants. All patients found VR to be a meaningful addition to their therapy sessions, with several wanting more exposure.	0/9 (0)	—^e^
Bouchard et al [52]	SSQ^f^, PQ^g^, and GPQ^h^	No significant increases in simulator sickness after exposure sessions (*P*>.20). Good level of presence that increased with a higher number of exposures.	2/17 (12)	Yes
Geraets et al [53]	—	VR treatment was well tolerated and deemed acceptable for most participants.	2/15 (13)	—
Hur et al [54]	—	—	16/73 (21)	—
Jeong et al [55]	—	—	52/115 (45)	—
Kampmann et al [56]	—	Simulator sickness led one patient to drop out.	5/20 (25)	Yes
Kim et al [47]	SSQ	Participants with SAD^i^ experienced significantly more simulator sickness than participants without SAD (*P*=.003).	2/54 (4)	—
Kim et al [57]	—	—	9/74 (12)	—
Kim et al [48]	SSQ	Low levels of simulator sickness.	3/24 (13)	—
Kovar [58]	—	—	0/10 (0)	—
Lindner et al [59]	NEQ^j^	High stress levels and low levels of satisfaction.	3/23 (13)	Yes
Moldovan and David [49]	ITQ^k^ and PQ	No moderating effect of immersion and presence on pre- and posttest anxiety.	0/32 (0)	—
Perandré and Haydu [60]	SPI^l^	High sense of presence reported by both participants.	0/2 (0)	—
Price and Anderson [61]	—	From a randomly selected subset of videotaped sessions (14%), high participant compliance was found, with 92% of the VR treatment protocol being completed.	0/33 (0)	—
Rubin et al [62]	—	—	2/21 (10)	—
Trahan et al [63]	SUS^m^	High usability reported by the participant.	0/1 (0)	—
Zainal et al [64]	NSQ, IPQ^n^, and SSQ	Acceptable presence and low levels of simulator sickness. High levels of homework compliance. Participants (85%) would recommend it to others with SAD. High levels of acceptability and usability.	9/44 (21)	—

^a^ITT: intention-to-treat analysis.

^b^CSQ: Client Satisfaction Questionnaire.

^c^NSQ: nonstandardized questions.

^d^HMD: head-mounted display.

^e^Not reported.

^f^SSQ: Simulator Sickness Questionnaire.

^g^PQ: Presence Questionnaire.

^h^GPQ: Gatineau Presence Questionnaire.

^i^SAD: social anxiety disorder.

^j^NEQ: Negative Effects Questionnaire.

^k^ITQ: Immersive Tendencies Questionnaire.

^l^SPI: Sense of Presence Inventory.

^m^SUS: System Usability Scale.

^n^IPQ: Igroup Presence Questionnaire.

### Quality Assessment Results

Multimedia Appendix 2 [47-64] contains a table of quality assessment results for the included studies. In all RCT studies [48,49,51,52,56,61,62,64], randomization was reported, but schedule details were unclear in 11% (2/18) of the studies [48,61]. All RCT studies reported comparable baseline group analyses. In total, 38% (3/8) of the RCT studies reported complete outcome data, which is defined as ≥80% [49,56,64]. All but the RCT studies by Kim et al [48], Moldovan and David [49], Price and Anderson [61], and Rubin et al [62] reported blinding of outcome assessors, which was applied at the pretest measurements. All RCT studies except those by Bouchard et al [52], Kampmann et al [56], and Rubin et al [62] reported that participants adhered to their assigned VR interventions.

In the quantitative descriptive studies [53,60,63], the sampling strategy was relevant to the research question except in 33% (1/3) of the studies, in which details were unclear [53]. All quantitative descriptive study samples were representative of the target population, and the measures fulfilled the inclusion criteria. Nonresponse bias was low in all studies except one (2/3, 67%) [60]. Statistical analyses were appropriate to answer the research question in 33% (1/3) of the studies [53] but unclear in the other 2 [60,63].

In the quantitative nonrandomized studies [47,54,55,57-59], participants were representative of the target population, measurements were appropriate regarding both the outcome and intervention, and there were complete outcome data (defined as ≥80%) in all but 2 studies (4/6, 67%) [54,55]. Confounds were accounted for in the design and analysis of 50% (3/6) of the studies [54,55,59]. In total, 67% (2/3) of the quantitative nonrandomized studies reported that the intervention was administered as intended [54,55].

In the single qualitative interview study [50], the qualitative approach was appropriate to answer the research question; the data collection methods were adequate to address the research question; findings were adequately derived from the data; the interpretation of the results was sufficiently substantiated by the data; and there was coherence between qualitative data sources, collection, analysis, and interpretation.

## Discussion

### Principal Findings

#### Overview

SAD is a common and debilitating anxiety disorder that affects occupational and social functioning [2]. Current in vivo–based exposure therapies require significant time, resources, and effort, which results in limited treatment dissemination [6]. VR technology provides an alternative modality for treating SAD [19]; however, contemporary evidence on the user experience of VR for SAD is sparse. This systematic review was conducted to provide a comprehensive and up-to-date account of the available evidence regarding the effectiveness and user experience (ie, safety, usability, acceptability, and attrition) of VR interventions for the treatment of SAD.

#### Effectiveness of VR Interventions for SAD

Our review found that VR interventions can effectively treat SAD in adult populations, which is congruent with the existing literature [6,20-24]. It is interesting to note that, although our search terms and inclusion criteria were open to any VR-based intervention for treating people with SAD (eg, providing relaxation, cognitive distraction, exposure therapy, and psychoeducation), all the included interventions were intended for exposure therapy. This indicates that VRET dominates the research field of VR-based interventions for SAD.

Studies including follow-up measures highlight the maintenance of SAD symptom improvement from 1 week [62] to 1 year [51], indicating that VRET can provide effective short- and long-term treatment for SAD symptoms. This is impressive given that the study showing maintained benefits for up to 1 year involved only 4 treatment sessions [51]. However, it is important to note that this study only included participants with a fear of public speaking as the primary social fear as opposed to other social situations (eg, going to dinner with friends), limiting the generalizability of the findings [65]. Nevertheless, our findings suggest that VRET can be a rapidly effective treatment for SAD with the potential to provide long-term symptom improvement.

#### Safety of VR Interventions for SAD

Simulator sickness was a common measure of safety in the reviewed studies. Participant simulator sickness was reportedly low in most studies. However, it was found that participants with an SAD diagnosis were more prone to simulator sickness when compared with participants without SAD in one study [47]. This could be because patients with anxiety tend to experience greater motion discomfort [66,67]. For example, patients with anxiety may be more susceptible to irregular breathing and hyperventilation, leading to dizziness and nausea when exposed to fear-inducing cues. This may exacerbate the body’s interpretation of disparities in visual and vestibular systems as possible deadly causes (ie, poison) and potentially lead to nausea and vomiting [25]. Another safety consideration is the absence of other physical injuries (eg, collisions with real-world objects, poor posture, headset discomfort, and seizures) reported in the reviewed studies, which supports VR as a safe SAD treatment.

However, although the research safety findings are encouraging, the limitations of these studies are important to note. For example, most studies screened out participants who were unable to tolerate the VR environment and HMD or those who had a history of seizures. This would result in a sampling bias in favor of VR safety. Furthermore, all studies except for one [63] were conducted in controlled settings (ie, hospitals and clinics) that were supervised by clinicians, further reducing risks that would otherwise be significant when using VR alone. For example, an individual purchasing and using a VR system at home may collide with real-world objects without the intervention of a third party. As such, more research is required to evaluate the safety of VR for SAD in nonclinical, unsupervised settings.

#### Usability of VR Interventions for SAD

There was large variability in the VR software used for SAD. This is likely due to the infancy of VR for SAD. With such variability, it is inevitable that reports of usability will vary according to the hardware and software used, with some programs being easier to use than others.

A distinct hindrance in evaluating the usability of VR for SAD was the lack of an existing framework. The studies largely used nonstandardized questions and qualitative feedback to determine usability, making it difficult to generalize findings across multiple studies. Although most studies did not comment on aspects of usability, those that did provided valuable information on the usability of VR for SAD. Studies in which practitioners delivered VR therapy to individual participants reported high levels of usability, such as the ease of setting up and navigating the hardware and software. However, reports of VR use in a group setting described low levels of usability, significant amounts of time spent on setting up and storing the equipment, and loss of focus on the exposure experience when therapists were helping others with their HMDs [50].

The differences in usability between individualized and group settings highlight important requirements for the use of VR interventions for SAD. Primarily, VR technology for SAD needs to be easy to learn by patients, and it is important that errors are limited in frequency and severity and that patients can recover from errors largely autonomously. As such, we propose a “VR usability framework” for the measurement of usability of VR for SAD that borrows elements from the usability heuristics by Nielsen [33]: (1) “learnability,” assessing how easy it is for a patient to set up and learn the VR technology; (2) “errors,” assessing the frequency, severity, and recoverability of errors autonomously by the patient; and (3) “memorability,” how easy it is to re-establish proficiency after a period of absence.

Using the VR usability framework, current trends show variability in the usability of VR for SAD. VR used in group therapy has a steep learning curve and requires substantial input from therapists to work through errors, and it is difficult to re-establish proficiency in it after a period of absence (eg, some participants wished they could take the equipment home) [50]. In contrast, VR used in individualized therapy is easy to learn, patients can autonomously handle errors, and they are familiar with the technology upon return [63,64]. Thus, VR may be more user-friendly in one-on-one therapy as opposed to a group setting, as articulated by the VR usability framework.

#### Acceptability of VR Interventions for SAD

The results we found regarding high VR acceptability in adult patients with SAD are congruent with earlier research by Saxena [35]. Empirical findings indicate that VR for SAD is generally acceptable to adult patients with SAD, with high scores on standardized measures of satisfaction reported by most patients. Positive qualitative responses suggest that VR allowed patients to gain more insights into their anxiety and a better understanding of the social situations that they would normally avoid or be too emotionally activated to observe [50]. For example, in real life, an individual with social anxiety may avoidantly play with their phone when someone sits next to them in a cafeteria rather than perceive the encounter as a valued learning experience. Therefore, it is likely that many adults with SAD who willingly undergo VR therapy will find the experience acceptable.

Conversely, one study [59] found that some patients reported that their expectations for the treatment were not fulfilled, and some reported feeling more stress during VR. It was also found that positive expectations of VR effectiveness as well as a positive working alliance with the therapist were significantly correlated with positive emotional changes [49]. Therefore, VR treatment may not be acceptable for all adults with SAD based on individual differences regarding their previous VR experience, their perceptions of VR therapy helpfulness, their level of distress tolerance to exposure to digital stimuli before habituation [68], and the nature of their relationship with the therapist offering VR treatment.

#### Attrition of VR Interventions for SAD

This review found that the attrition rate across most studies was relatively low and within acceptable levels (≤20%) [46]. Indeed, attrition rates for the use of VR interventions for SAD were found to be substantially lower than estimates from VRET in anxiety disorders [43]. Considering this, it appears that patients with SAD continue with treatment more than other patients with anxiety.

There may be several reasons for the low average attrition rate finding. First, patients with SAD may prefer to learn more about social situations in a VR space. An individual with SAD may be curious about learning about social situations but may struggle to overcome the anxiety associated with placing themselves in an environment where negative evaluation is possible. By engaging with VR, patients with SAD have the knowledge that they can exit the simulation at any point, giving them the opportunity to learn about social situations without real-world social consequences. Second, patients with SAD may be more tolerant of the potentially negative effects of VR (eg, simulator sickness) when compared with the general population with anxiety [43]. Third, patients with SAD may be more hesitant to drop out of therapy for fear of negative evaluation by examiners. For instance, patients with SAD may be more likely to remain in a study because of social desirability bias—the tendency to respond in a certain way to avoid criticism [69].

It is important to note that the observed attrition rates are heterogeneous. Some studies reported proportionally higher attrition rates than others [54,55]. This may be due to the differences in the number of sessions involved in different studies. For example, some studies were composed of single sessions [49,59,62], whereas the study with the highest attrition had 9 to 17 sessions [55]. As it takes longer to deliver all sessions, there is more opportunity for participants to drop out. Furthermore, attrition was defined in this review as those who did not complete measurements during or after intervention use, including completion of follow-up measures. Considering that some studies included follow-up measures of 3 months after the intervention or longer, it is plausible that participants may not have re-engaged in these measures for several reasons. These could potentially include both therapy-related factors (eg, intolerance of VR-induced anxiety, simulator sickness, and low satisfaction levels) or factors outside of therapy (eg, moving away, becoming too busy in everyday life, and major life events).

### Recommendations

#### Safety

With regard to safety, the primary issue identified in this review was simulator sickness. Several factors appear to be related to the susceptibility to simulator sickness. If simulator sickness is exacerbated by physiological symptoms of anxiety (eg, hyperventilation leading to dizziness) [66,67], it may be helpful to target these symptoms with clinical treatment before using VR technology. This is in line with other studies exploring attrition in anxiety disorders [43], which found that VRET attrition occurs early in treatment because of factors such as dizziness. As such, VR protocols for SAD should aim to improve retention at the beginning of treatment using a phase-based approach that includes strategies to tolerate negative emotions before immersion in VR. These may include implementation of relaxation and grounding techniques [70] or the prescription of antinausea medications. Changes in VR technology can also be applied to reduce simulator sickness [48]. Blurring or lowering the resolution of a VR image has been shown to reduce simulator sickness and improve the sense of reality [71,72].

#### Future Research

Presently, there are many research gaps in the literature regarding the user experience of VR for SAD. The development of a standardized measure to assess the usability of VR for SAD has the potential to identify prominent issues with usability and aid in the development of future VR programs. This measure may include elements identified in the VR usability framework discussed previously to assess learnability, error recoverability, and memorability. This may be applied to technical developments in VR that would likely improve VR’s “plug-n-play” capability for SAD and other anxiety disorder treatments. Future research should also delve deeper into the study of simulator sickness in patients with SAD when compared with both healthy controls and patients with other anxiety disorders. This may lead to valuable information on reducing simulator sickness, thereby reducing the levels of attrition and improving the user experience of VR for SAD. Finally, there were no studies found that specifically targeted a child or adolescent population. Given that the onset of SAD typically occurs around adolescence [1], future studies should evaluate the efficacy of early intervention of VR for SAD, particularly given adolescents’ success with VR for psychological distress [32].

### Limitations

This review has several limitations. First, our review did not perform a cost-benefit analysis of the hardware identified (eg, HMDs). Affordability could have implications for the acceptability of VR among consumers with SAD. Second, we included only English-language studies, and there may have been pertinent articles published in other languages. Third, this study conducted a qualitative review of studies with different designs. Although the MMAT [46] was used to assess the quality of the studies, there is still a risk of subjective reviewer bias in addressing its criteria. Finally, studies may have been missed in our search because of obscure nomenclature (eg, research publications that did not clearly specify the use of a VR intervention for SAD in their title and abstract).

### Conclusions

Our review findings showed that VRET interventions can generally provide an effective, safe, usable, acceptable, and low-attrition treatment option for adults with SAD. Nevertheless, there are research gaps evident when appraising user experience outcomes. These include the need to conduct more VR research with children and adolescents with SAD. We also do not yet know the specific causes of elevated simulator sickness in patients with SAD compared with participants without SAD or how effective other VR-based interventions beyond exposure therapy (eg, focused on mindfulness, relaxation, or cognitive distraction) are in the treatment of SAD. Further experimental studies (eg, pilot feasibility studies and RCTs) are required to explore these domains.

## Appendix

Multimedia Appendix 1 PRISMA (Preferred Reporting Items for Systematic Reviews and Meta-Analyses) 2009 checklist.

Multimedia Appendix 2 Quality assessment results of the included studies.

## Abbreviations

CAVE: cave automatic virtual environment
HMD: head-mounted display
MMAT: Mixed Methods Appraisal Tool
PRISMA: Preferred Reporting Items for Systematic Reviews and Meta-Analyses
RCT: randomized controlled trial
SAD: social anxiety disorder
VR: virtual reality
VRET: virtual reality exposure therapy
