# Supplementary material for: Effectiveness and User Experience of Virtual Reality for Social Anxiety Disorder: Systematic Review
Source: JMIR Ment Health. 2024 Feb 8;11:e48916. doi: 10.2196/48916 (PMC10884902; doi:10.2196/48916)
Supplement: Multimedia Appendix 2 [file mental_v11i1e48916_app2.pdf]

Multimedia Appendix. Quality Assessment Results of Included Studies Using the Mixed Methods Appraisal Tool (MMAT) version 2018

**Category of Study Designs**	**Methodological Quality Criteria**	**Anderson et al [51]**	**Arnfred et al [50]**	**Bouchard et al [52]**	**Geraets et al [53]**	**Hur et al [54]**	**Jeong et al [55]**	**Kampmann et al [56]**	**Kim et al [47]**	**Kim et al [57]**	**Kim et al [48]**	**Kovar [58]**	**Lindner et al [59]**	**Moldovan and Price [49]**	**Perandré and Haydu [60]**	**Price and Anderson [61]**	**Rubin et al [62]**	**Trahan et al [63]**	**Zainal et al [64]**
Screening questions	S1. Are there clear research questions?	Yes	Yes	Yes	Yes	Yes	Yes	Yes	Yes	Yes	Yes	Yes	Yes	Yes	Yes	Yes	Yes	Yes	Yes
	S2. Do the collected data allow to address the research questions?	Yes	Yes	Yes	Yes	Yes	Yes	Yes	Yes	Yes	Yes	Yes	Yes	Yes	Yes	Yes	Yes	Yes	Yes
Qualitative	1.1. Is the qualitative approach appropriate to answer the research question?	N/A	Yes	N/A	N/A	N/A	N/A	N/A	N/A	N/A	N/A	N/A	N/A	N/A	N/A	N/A	N/A	N/A	N/A
	1.2. Are the qualitative data collection methods adequate to address the research question?	N/A	Yes	N/A	N/A	N/A	N/A	N/A	N/A	N/A	N/A	N/A	N/A	N/A	N/A	N/A	N/A	N/A	N/A
	1.3. Are the findings adequately derived from the data?	N/A	Yes	N/A	N/A	N/A	N/A	N/A	N/A	N/A	N/A	N/A	N/A	N/A	N/A	N/A	N/A	N/A	N/A
	1.4. Is the interpretation of results sufficiently substantiated by data?	N/A	Yes	N/A	N/A	N/A	N/A	N/A	N/A	N/A	N/A	N/A	N/A	N/A	N/A	N/A	N/A	N/A	N/A
	1.5. Is there coherence between qualitative data sources, collection, analysis and interpretation?	N/A	Yes	N/A	N/A	N/A	N/A	N/A	N/A	N/A	N/A	N/A	N/A	N/A	N/A	N/A	N/A	N/A	N/A
2. Quantitative randomized controlled trials	2.1. Is randomization appropriately performed?	Yes	N/A	Yes	N/A	N/A	N/A	Yes	N/A	N/A	Can’t tell	N/A	N/A	Yes	N/A	Can’t tell	Yes	N/A	Yes
	2.2. Are the groups	Yes	N/A	Yes	N/A	N/A	N/A	Yes	N/A	N/A	Yes	N/A	N/A	Yes	N/A	Yes	Yes	N/A	Yes

	comparable at baseline?																		
	2.3. Are there complete outcome data?	No	N/A	No	N/A	N/A	N/A	Yes	N/A	N/A	No	N/A	N/A	Yes	N/A	Can’t tell	No	N/A	Yes
	2.4. Are outcome assessors blinded to the intervention provided?	Yes	N/A	Yes	N/A	N/A	N/A	Yes	N/A	N/A	Can’t tell	N/A	N/A	Can’t tell	N/A	Can’t tell	No	N/A	Yes
	2.5. Did the participants adhere to the assigned intervention?	Yes	N/A	Can’t tell	N/A	N/A	N/A	Can’t tell	N/A	N/A	Yes	N/A	N/A	Yes	N/A	Yes	Can’t tell	N/A	yes
3. Quantitative nonrandomized controlled trials	3.1. Are the participants representative of the target population?	N/A	N/A	N/A	N/A	Yes	Yes	N/A	Yes	Yes	N/A	Yes	Yes	N/A	N/A	N/A	N/A	N/A	N/A
	3.2. Are measurements appropriate regarding both the outcome and intervention (or exposure)?	N/A	N/A	N/A	N/A	Yes	Yes	N/A	Yes	Yes	N/A	Yes	Yes	N/A	N/A	N/A	N/A	N/A	N/A
	3.3. Are there complete outcome data?	N/A	N/A	N/A	N/A	No	No	N/A	Yes	Yes	N/A	Yes	Yes	N/A	N/A	N/A	N/A	N/A	N/A
	3.4. Are the confounders accounted for in the design and analysis?	N/A	N/A	N/A	N/A	Yes	Yes	N/A	Can’t tell	No	N/A	Can’t tell	Yes	N/A	N/A	N/A	N/A	N/A	N/A
	3.5. During the study period, is the intervention administered (or exposure occurred) as intended?	N/A	N/A	N/A	N/A	Yes	Yes	N/A	No	Can’t tell	N/A	Can’t tell	No	N/A	N/A	N/A	N/A	N/A	N/A
4. Quantitative descriptive	4.1. Is the sampling strategy relevant to address the research question?	N/A	N/A	N/A	Can’t tell	N/A	N/A	N/A	N/A	N/A	N/A	N/A	N/A	N/A	Yes	N/A	N/A	Yes	N/A
	4.2. Is the sample representative of the target population?	N/A	N/A	N/A	Yes	N/A	N/A	N/A	N/A	N/A	N/A	N/A	N/A	N/A	Yes	N/A	N/A	Yes	N/A

	4.3. Are measurements appropriate?	N/A	N/A	N/A	Yes	N/A	N/A	N/A	N/A	N/A	N/A	N/A	N/A	N/A	Yes	N/A	N/A	Yes	N/A
	4.4. Is the risk of nonresponse bias low?	N/A	N/A	N/A	Yes	N/A	N/A	N/A	N/A	N/A	N/A	N/A	N/A	N/A	No	N/A	N/A	Yes	N/A
	4.5. Is the statistical analysis appropriate to answer the research question?	N/A	N/A	N/A	Yes	N/A	N/A	N/A	N/A	N/A	N/A	N/A	N/A	N/A	Can’t tell	N/A	N/A	Can’t tell	N/A
5. Mixed methods	5.1. Is there an adequate rationale for using a mixed methods design to address the research question?	N/A	N/A	N/A	N/A	N/A	N/A	N/A	N/A	N/A	N/A	N/A	N/A	N/A	N/A	N/A	N/A	N/A	N/A
	5.2. Are the different components of the study effectively integrated to answer the research question?	N/A	N/A	N/A	N/A	N/A	N/A	N/A	N/A	N/A	N/A	N/A	N/A	N/A	N/A	N/A	N/A	N/A	N/A
	5.3. Are the outputs of the integration of qualitative and quantitative components adequately interpreted?	N/A	N/A	N/A	N/A	N/A	N/A	N/A	N/A	N/A	N/A	N/A	N/A	N/A	N/A	N/A	N/A	N/A	N/A
	5.4. Are divergences and inconsistencies between quantitative and qualitative results adequately addressed?	N/A	N/A	N/A	N/A	N/A	N/A	N/A	N/A	N/A	N/A	N/A	N/A	N/A	N/A	N/A	N/A	N/A	N/A
	5.5. Do the different components of the study adhere to the quality criteria of each tradition of the	N/A	N/A	N/A	N/A	N/A	N/A	N/A	N/A	N/A	N/A	N/A	N/A	N/A	N/A	N/A	N/A	N/A	N/A

	methods involved?	

Note. This is a Multimedia Appendix to a full manuscript published in JMIR Mental Health. For full copyright and citation information see http://dx.doi.org/10.2196/jmir.48916.
